# Sex- and age-specific differences in excess mortality in China during the COVID-19 pandemic: a study based on official vital statistics data

**DOI:** 10.3389/fepid.2025.1595453

**Published:** 2025-09-25

**Authors:** Yao Li

**Affiliations:** CriDIS, Department of Sociology, Catholic University of Louvain, Louvain-la-neuve, Belgium

**Keywords:** excess mortality, excess mortality rate, age-and sex-specific excess mortality, COVID-19, zero-COVID

## Abstract

**Introduction:**

This study aimed to investigate the excess mortality observed in China between 2020 and 2023 and its underlying causes, including the COVID-19 pandemic, sex ratio, and aging trends.

**Methods:**

We collected data regarding number of deaths and mortality rates of the years 2015–2019 from the China Statistical Yearbook of Population and Employment, and analyzed the data by age and sex. We created a standardized sex and age structure dataset and compared the excess mortality rates of different sexes and age groups for the years 2020 to 2023.

**Results:**

The sex-and age-specific number of deaths in all three years decreased compared to the number of deaths in the standardized sex and age structure dataset. The most significant decline was observed in 2021, whereas the number of deaths in 2022 and 2023 increased compared to that in 2021. It has been found that excess mortality is generally less prevalent among older age groups, and excess mortality rate tends to be lower among younger age groups. Additionally, in 2021, 2022, and 2023, the excess mortality rate was greater for men than for women, whereas it was greater for women in 2020. These differences can be attributed to various factors.

**Discussion:**

This is the first study to examine excess mortality in China during the COVID-19 pandemic using age- and sex-standardized data. These findings underscore the need for a deeper exploration of the effects of sex and age on health outcomes.

## 1 Introduction

Between 2020 and 2023, China faced unprecedented challenges posed by the COVID-19 pandemic. This global health crisis imposed immense pressure on China's public health system and exerted profound impacts on the society, economy, and population health. On December 7, 2022, China's public health authorities issued the “New Ten Measures”, significantly relaxing COVID-19 control measures. This was followed by the formal downgrade of COVID-19 from a Category A to a Category B infectious disease on January 8, 2023, and the reopening of national borders (1). Between December 15 and 31, 2022, China experienced a rapid and widespread outbreak of the Omicron variant, with an estimated daily infection rate of 0.42%, meaning the number of infections doubled approximately every 1.6 days (2). In Sichuan Province, the peak of infections occurred between December 12 and 23, with a basic reproduction number (R_0_) estimated at 4.15 (3). It is estimated that approximately 90% of the population—over one billion people—were infected during this two-week period, resulting in what has been termed a “compressed epidemic.” Modeling from Shanghai projected that at the epidemic's peak, demand would exceed 74,000 hospital beds and 3,700 ICU beds, far surpassing the existing healthcare system's capacity. The sudden and comprehensive lifting of restrictions led to a surge in the effective reproduction number (R_t_) to 3.44, overwhelming the healthcare system's buffer capacity. As a result, patients suffering from non-COVID critical conditions—such as cardiovascular and cerebrovascular diseases—faced delayed treatment due to medical resource shortages, contributing to an increase in indirect excess mortality (4). Therefore, studying excess mortality in China is crucial. Excess mortality, which is the difference between actual and expected deaths, is a crucial metric for evaluating the impact of emergencies, such as pandemics, on population health.

Data regarding death during the COVID-19 pandemic in China have been questioned by many researchers. Some argue that the increased prevalence of mental illness due to lockdown measures and logistical barriers may also have contributed to excess mortality. Moreover, China's sex ratio and aging trends are significant factors that influence excess mortality. From 1980–2016, male individuals constituted a higher proportion of those born, indicating a high sex ratio at birth and in the population. However, the sex ratio was relatively lower before 1980 and after 2016. At the family level, the sex ratio rapidly increases with increasing birth order, with a particularly high sex ratio among the last-born children. The strict fertility policy implemented in China in 1980 resulted in a continuously increasing sex ratio at birth, leading to a significant number of missing girls (5). Possible adjustments to China's fertility policy have been discussed along with measures that could lead to a more normal sex ratio at birth. Chi et al. (2013) investigated current attitudes towards sex preferences among people of reproductive age in China to better understand the persistently high sex ratio (6). The study found that, while preference for a son weakened considerably in the current reproductive generation, it did not completely disappear. In addition, disparities in mortality rates between sexes exist. Evidence of this disparity continues to emerge as the number of COVID-19 cases increases. According to the African Population and Health Research Centre (APHRC), statistics on COVID-19 outbreaks vary considerably around the world. However, male patients are generally 20% more likely to be hospitalized than female patients. Once hospitalized, males are more likely to require intensive care and may experience fatal outcomes (7).

The elderly population is a high-risk group during a pandemic, and the accelerated aging population in China in recent years has been a crucial factor contributing to excess mortality. Hou (2019) explored the nature and causes of China's aging population, the lackluster effects of the universal two-child policy, and the uniqueness of China's aging (8). After experiencing a population boom, China is now facing the challenge of an aging population, which will have a significant impact on the economy, society, and population. According to Bai et al. (2020), China is expected to experience the largest wave of population aging over the next 30 years, with a high number of oldest-old individuals, high number of empty-nest elderly individuals, and high elderly dependency. Despite advances in medical technology and treatments, the COVID-19 pandemic continues to have a severe impact on population health (9). Zhang et al. (2020) identified the risk factors associated with COVID-19 severity and mortality in patients with severe disease using multivariate logistic regression analysis, which revealed that older age and a higher number of affected lung lobes influenced death of patients with COVID-19 in Wuhan, China. COVID-19-related deaths among older adults have significantly affected life expectancies in most countries (10).

The conventional method involves calculating the average population deaths over a five-year period and subtracting this from the 2022 data to determine excess mortality and excess mortality rates. If China's excess mortality between 2015 and 2022 were examined using the traditional method, the rise in life expectancy would be accompanied by high excess mortality. This is because the traditional method does not consider the accelerated aging process in China or the different sex-structured population at different ages. Over the past decade, age-specific mortality rates in China have steadily declined, and average life expectancy has continued to rise—an outcome shaped by a combination of factors. These improvements are primarily attributed to advances in medical care and public health, though they are also influenced by broader social, economic, policy, and technological developments. As China's economy has progressed and lifestyles have changed, the country's disease profile has shifted from one dominated by infectious diseases to one characterized by chronic non-communicable diseases (NCDs). Cardiovascular diseases, diabetes, respiratory illnesses, and cancers have emerged as the leading causes of death. Although these NCDs accounted for 90.1% of deaths in 2019, their mortality rates have significantly declined. For instance, the mortality rate from cardiovascular diseases decreased by 59.5%, and cancer-related deaths also showed a downward trend (11). Moreover, China has made notable progress in early screening for chronic conditions, public health education, and lifestyle interventions. The management rates for hypertension and diabetes have consistently improved, and public awareness of health risks has grown substantially (12). Adjusting and estimating excess mortality according to sex and age can help overcome this limitation.

This study aimed to investigate the excess mortality in China in 2020, 2021, 2022 and 2023 and its underlying causes, including the COVID-19 pandemic, sex ratio, and aging trends. After controlling for these factors, we estimated the excess mortality toll and excess mortality rate in China to gain a more comprehensive understanding. Our findings provide valuable references and insights for future public health policy formulations, epidemic prevention, and control efforts.

## 2 Literature review

When examining excess mortality data in China during the COVID-19 pandemic, it is important to review the evolution of excess mortality calculation methods and their applicability in different contexts. The accuracy and scientific validity of the calculation method for excess mortality, as an important indicator of the impact of the epidemic on society's health, are crucial for assessing the severity of the epidemic. Standardization of the treatment of mortality data is crucial to ensure the comparability and reliability of the study results. Adjusting for factors such as age and sex can more accurately reveal changes in mortality risk among different populations during an epidemic. Furthermore, it is important to consider research conducted by scholars, both domestically and internationally, on excess mortality in China to gain a comprehensive understanding of the impact of COVID-19 on the health of the Chinese society. This literature review begins by examining the calculation method for excess mortality and the standardization of death data. Relevant studies from both domestic and international sources have been analyzed to provide a theoretical foundation and reference basis for subsequent in-depth studies. Although it is not yet possible to estimate the excess mortality associated with COVID-19 in China, the high infection rate and relatively low immunity level of the Chinese population will likely result in excess mortality and a reduction in life expectancy. A review of China's life expectancy trends will be necessary once excess mortality data become available in the near future.

Modig et al. (2020) estimated age- and sex-specific death rates and sex-based differences in death rates in Sweden to obtain more accurate excess mortality estimates attributed to COVID-19. Current excess mortality results in a decline in the remaining life expectancy by three years for men and two years for women (13). Vestergaard et al. (2020) provided preliminary pooled estimates of all-cause mortality in 24 European countries/federal states participating in the European Monitoring of Excess Mortality for Public Health Action (EuroMOMO) network from March 2020 to April 2020. The study found that excess mortality primarily affected individuals aged 65 years and older, accounting for 91% of all excess mortality, but also affected 8% of individuals aged 45–64 years and 1% of those aged 15–44 years (14). Karlinsky et al. (2021) collected all-cause mortality data from 94 countries and territories on weekly, monthly, or quarterly bases, which are openly available as a regularly updated World Mortality Dataset and demonstrated that excess mortality, which is the increase in all-cause mortality compared with expected mortality, is widely considered a more objective indicator of the COVID-19 death toll (15). Dorrucci et al. (2021) aimed to compare all-cause excess mortality during two waves that occurred in 2020 using nationwide data. Negative binomial models with time modeled by quadratic splines were used to estimate all-cause excess mortality (16).

The DSP system documented 580,819 deaths between January 2020 and March, 2020. The total mortality rate observed in the three Wuhan DSP districts was 56% higher than predicted. This increase was mainly due to a significant increase in deaths related to COVID-19, as well as a more modest increase in deaths from other diseases, such as cardiovascular disease and diabetes. During the three months of the COVID-19 outbreak in other parts of China, there was no increase in overall mortality, except in Wuhan. The lower death rates from certain non-COVID-19 related diseases during the lockdown may be attributed to associated behavioral changes (17). Between 2020 and 2021, China officially reported 4,820 COVID-19 deaths. However, academic estimates place the number of excess deaths at approximately 17,900 (ranging from 7,540–30,100), roughly 3.7 times higher than the reported figure. Despite this, China's estimated excess mortality rate remains significantly below the global average of 120.3 per 100,000 population, and also lower than the East Asian regional average of 0.5 per 100,000. Notably, these estimates are derived entirely from modeling, rather than from a comprehensive national death registration system. The absence of publicly available, nationwide mortality data introduces a high degree of uncertainty to these projections (18).

Cai et al. (2022) published a study that modeled an excess of 1.5 million deaths due to COVID-19 if China ended its zero-COVID strategy with its then current vaccination status. Another cohort study analyzed obituary data from three universities in China (two in Beijing and one in Heilongjiang) and search engine data from the Baidu Index in each region of China from January 1, 2016, to January 31, 2023. This study extrapolated the relative increase in mortality in Beijing and Heilongjiang to the rest of China to calculate region-specific excess mortality, with an estimated 1.87 million excess mortalities occurring among individuals aged 30 years and older in China. Excess mortality was mainly observed among older individuals and was present in all provinces of mainland China, except for Tibet (19). A cohort study conducted in China found that the sudden lifting of the zero COVID policy was associated with a significant increase in all-cause mortality (20). Liu (2023) calculated the excess mortality and death rates for each province based on the population and death rate data from 2015–2022. The baseline population, deaths, and death rates for the 31 provinces were derived using the averages of the 2015–2019 data. China's excess mortality exceeds one million annually and reached over four million by the end of 2022. The percentage of excess mortality increased in all regions of China from 2020–2022, with the eastern region experiencing the largest increase of over 88%. A nationwide increase of >50% was observed (21, 22).

To date, studies on excess mortality associated with the COVID-19 pandemic in China have employed a diverse range of methodologies. These include time-series analyses based on official death registries (17, 23), the use of university obituary data and online search indices (24), measurements of online mourning-related search volume (25), as well as mathematical infectious disease modeling (26). Research indicates that in early 2020, excess mortality was significantly elevated in Wuhan (56%), while no substantial nationwide increase was observed in other regions of China (17). During the post-zero-COVID reopening phase in late 2022, scholars employing obituary data and large-scale digital monitoring inferred that nationwide excess deaths may have ranged from 0.7–1.9 million (24–26). Compared to countries such as the United States, European nations, South Korea, and India, China's excess mortality rate prior to the lifting of its COVID-19 restrictions remained at a moderate to low level (24). However, the discrepancy between these estimates and the official death toll is substantial, primarily due to limitations in data transparency and assumptions embedded in various modeling approaches. These challenges underscore the urgent need for the public release of nationally representative, age-disaggregated death registration data in order to validate and refine current excess mortality estimates. Ioannidis et al. estimated the total number of COVID-19 deaths in mainland China from December 7, 2022—the date marking the end of the country's “zero-COVID” policy—through the summer of 2023. Drawing on empirical infection fatality rates (IFRs) observed during Omicron waves in Hong Kong and South Korea, the study employed age-stratified calculations and incorporated data from long-term care facilities. The findings suggest that if the entire population were infected, the estimated death toll in mainland China could range from approximately 987,455 (based on the Hong Kong model) to 619,549 (based on the South Korea model). The authors critically noted that China's official reporting criteria for COVID-19 deaths were extremely narrow, counting only deaths directly attributed to COVID-19 with a primary diagnosis of pneumonia. As a result, the study contends that a substantial number of actual deaths were likely omitted from official tallies, pointing to the likelihood of significant underreporting (27). Owing to the limited availability of data in China, few studies have examined excess mortality during the COVID-19 pandemic, particularly in relation to excess mortality data in 2022 and 2023. To estimate excess mortality, existing data have been analyzed through unconventional means, such as the Baidu Index. However, official scientific sampling data have not yet been utilized for this estimation.

Campbell & Gunia (2020) reported that since late 2019, China repeatedly adjusted the criteria for identifying “confirmed cases” of COVID-19. These shifting definitions rendered early data difficult to compare over time, resulting in significant statistical volatility and undermining efforts to accurately track trends in infections and deaths. Moreover, the official reporting protocol only included deaths that occurred after hospital confirmation, meaning that many individuals who died at home or failed to seek timely medical attention may have been excluded from the official statistics (28). A modeling study by the University of Hong Kong estimated that by February 20, 2020, the actual number of infections in China may have been more than three times the official figures, and in Wuhan, the true COVID-19 death toll could have been as much as 20 times higher than reported (29). These discrepancies are partly attributed to the Chinese government's use of pandemic control as a tool of political propaganda, aiming to emphasize the success of its “zero-COVID” approach and its capacity to maintain control. In this context, there was institutional incentive to underreport both infections and deaths. Romaniuk and Burgers (2020) similarly argue that China's top leadership tends to reward favorable data, giving local officials strong motivation to understate the severity of the outbreak to gain positive evaluations from higher authorities. This mechanism of “reporting good news upwards” creates systemic risk of data suppression. During the early phase of the pandemic, whistleblowing doctors in Wuhan—including Dr. Li Wenliang—faced censorship, and local authorities reportedly hesitated to report the crisis to Beijing without explicit approval, indicating the presence of information control and delayed disclosure (30).

China's COVID-19 statistics have also been criticized for lacking transparency in terms of methodology, with no access to raw data, detailed explanations of definitions, or interim reports, making it difficult to assess their reliability. Armstrong (2023), citing WHO's Executive Director of Health Emergencies Michael Ryan, noted that China adopted an “extremely narrow” definition of COVID-19 deaths, only counting those who died of respiratory failure caused directly by the virus and whose cases were confirmed in hospitals. This excluded individuals who died in community settings or from underlying health conditions exacerbated by COVID-19. After the relaxation of the zero-COVID policy, hospitals and funeral homes experienced an overwhelming surge in patients and bereaved families, yet this suspected record-high mortality was still not reflected in the official data (31).

The China Statistical Yearbook of Population and Employment provides detailed data regarding death that are used to estimate the death status of the total population. This estimation can compensate for the lack of research on excess mortality during the COVID-19 pandemic in China. Importantly, this estimation is based solely on available data and may not be entirely accurate.

We found relatively few academic studies that critically examine the statistical results of sampling surveys conducted by China's National Bureau of Statistics (NBS), particularly with regard to mortality data. However, we did identify a number of systematic discussions focusing on the discrepancies between the total fertility rate (TFR) figures published by the NBS and those released by the National Health Commission (NHC, formerly the National Health and Family Planning Commission), as well as broader concerns about the overall reliability of these datasets.

Scholars generally consider the NBS data to be more scientifically robust, as it is based on the summation of age-specific fertility rates derived from large-scale, nationally representative household sampling surveys conducted annually. In contrast, the NHC's fertility statistics are drawn primarily from hospital birth records, which exclude mobile populations, out-of-wedlock births, and unregistered newborns. These omissions are typically not adjusted for through weighting or statistical correction, resulting in a systematic overestimation of fertility rates (32).

As the administrative body responsible for family planning policies, the NHC has long faced political pressures—whether to maintain stable birth rates or to support liberalization measures—and thus has had incentives to overstate fertility rates in order to demonstrate policy effectiveness or to assuage public concern (33). The NBS, by contrast, is considered relatively neutral. Nevertheless, its data may also be affected by local governments' reporting biases. In some cases, local authorities underreport births to avoid penalties under family planning regulations, potentially leading to an underestimation of TFR (34).

Overall, the NBS and NHC operate under two distinct statistical systems. The NBS's data is generally viewed as more neutral, with weaker incentives to obscure the truth and less political pressure. Conversely, the NHC's data is more likely to reflect institutional motivations to conceal or distort outcomes. Moreover, the age-disaggregated mortality data provided by the NBS is not classified by cause of death, which allows us to use all-cause mortality in estimating excess deaths without the bias introduced by differing definitions or classifications of COVID-19-related deaths. Since the China Population and Employment Statistical Yearbook is based on the NBS's sample survey data, which is relatively reliable, we have chosen to use this dataset for our statistical analysis.

## 3 Materials and methods

The data extracted from the China Statistical Yearbook of Population and Employment published by the China Statistics Press were processed and analyzed using Excel software. This yearbook compiles age-specific mortality data derived from the 1‰ population sample survey (covering the years 2016–2019, as well as 2021, 2022, and 2023) and the quinquennial 1% population sample survey (for 2015 and 2020). The mortality data cover the deaths of individuals of all sexes within each age group, from ages 0–89 years, and those aged 90 years and older. The annual statistical results reflect data up to November 1st of that year, indicating that the 2022 data included deaths from November 1, 2021, to October 31, 2022. As the COVID-19 outbreak in China occurred after the implementation of revised epidemic prevention measures announced on November 11, 2022, the 2022 data provides a detailed examination of the impact of lockdowns. The 2023 data offer insights into the manifestation of COVID-19 outbreaks and the surge capacities of healthcare systems in terms of excess mortality figures following the lifting of restrictions related to the disease. The 2024 Yearbook, published in December 2024, presents the most recently available data reflecting age-specific mortality patterns from November 2022 to October 2023.

To create a standardized sex- and age-structure dataset, we excluded data from 2020–2023 because of the significant impact of the COVID-19 pandemic and the associated mortality fluctuations caused by the virus and lockdown measures in China. We used data from the five years prior to the pandemic, specifically from 2015–2019, which included age- and sex-specific population and mortality data. Therefore, three datasets were used to standardize the data for 2020–2023—two sets of age-specific population data from 2019–2021, one from each year, and the other dataset comprising age- and sex-specific population data and mortality data from the five years prior to the start of COVID-19, i.e., the 2015–2019 period—to calculate the number of people at each age in each of these years with the mortality rates for each of these years to derive a standardized sex and age structure dataset.

Standardizing the age-specific mortality data for 2020, 2021, 2022, and 2023 is a crucial step in eliminating the influence of interannual population structure changes on mortality data. The use of a standardized sex- and age-structure dataset allowed for the comparison of mortality patterns across different years within the same age and sex groups. This effectively eliminated the confounding effects of age-structure variations and sex imbalances. Excess mortality was defined as the difference between the actual number of deaths and the expected number based on standardized sex and age structures. To calculate the excess mortality rate for each year, we compared the age-standardized mortality data with the expected deaths from standard sex and age structures using the following formula:

$$\begin{array}{rl} & Excess\;Mortality\;Rate\\ & =\;\frac{\begin{array}{rl} \;[ & \left(Sex-and\;Age-Standardized\;Deaths\right)\\ & \;-(Sex-and\;Age-Structured\;Dataset\;Deaths)] \end{array}}{Standard\;Sex-and\;Age-Structure\;Dataset\;Deaths}\times 100\text{\%} \end{array}$$

This study aimed to comprehensively and accurately analyze the changes in excess mortality rates in China from 2020–2023, providing robust data support for understanding the impact of the COVID-19 pandemic on the health of the Chinese population. The population and mortality data provided in the 2015–2022 China Statistical Yearbook of Population and Employment are based on sample sizes of approximately 1‰ or 1% of the population each year. There may have been some bias or fluctuations in sample size. Notably, the sample size in 2015 comprised approximately 1% of the entire population. The sample size in 2016 was insufficient to directly determine the mean value as it represented only 1‰ of the entire population. Therefore, we calculated the number of people of each age and sex by multiplying the total population in that year by the proportion of the sample size for each age group in the total sample size. The final result may deviate from the data of the China Population Yearbook, but is consistent with the data of the China Statistical Yearbook of Population and Employment.

Due to the fact that each yearbook's data pertains to the period from November of the preceding two years to October of the preceding year, for instance, the 2023 yearbook documents data for the period beginning from November 2021–October 2022, we henceforth refer to the 12-month data recorded in the 2023 yearbook as “data from 2022” for brevity.

## 4 Results

Age- and sex-specific demographic data from 2019 were used to calculate mortality figures and excess mortality rates for the years 2020–2023. These values were subsequently compared with the 2019 age- and sex-specific data. The study revealed that in comparison with 2019, the age- and sex-specific mortality in China decreased by approximately 10%, 34%, 22%, and 13% in 2020, 2021, 2022, and 2023, respectively. This decline can be attributed to the implementation of public health measures, including lockdowns, widespread mask wearing, and universal vaccination against the virus. However, a modest rebound was observed in the 2022 data compared with the 2021 data, albeit still significantly lower than the figures for both 2019 and 2020. In 2023, a more substantial rebound in mortality figures compared to those in 2021 and 2022 occurred, bringing it closer to the 2020 excess mortality data, but still markedly below the 2019 mortality data.

Following the processing of the data using the sex- and age-specific structure from 2015–2019 data, it was determined in Table 1 that the adjusted mortality counts for the years 2020, 2021, 2022, and 2023 were 7,162,906, 5,310,044, 6,248,920, and 6,927,452, respectively. All four figures are substantially lower than the expected mortality count based on standardized sex and age structure (7,622,949). The mortality rate in 2021, following standardization, was the lowest among these years. The excess mortality rates for all four years, adjusted using the standardized sex and age structure based on the standard life table, were 3%–4% lower than the excess mortality rate adjusted for age and sex in 2019. This finding suggests that China may have experienced a relatively high mortality rate in 2019.

**Table 1:** Sex- and age-standardized excess mortality data of 2020–2023.

Year	Number of deaths adjusted for 2019 data	Excess mortality rate adjusted for 2019 data	Number of deaths adjusted for standard life table data	Excess mortality rate adjusted for standard life table data
2019.11–2020.10	76,91,892 (76,86,456, 76,97,327)	−10.72% (−10,78%, −10,66%)	71,62,906 (71,57,660, 71,68,152)	−6.03% (−6,06%, −5,92%)
2020.11–2021.10	56,96,276 (56,91,598, 57,00,954)	−33.88% (−33,94%, −33,83%)	53,10,044 (53,05,527, 53,14,561)	−30.34% (−30,37%, −30,25%)
2021.11–2022.10	67,19,599 (67,14,518, 67,24,680)	−22.01% (−22,07%, −21,95%)	62,48,920 (62,44,020, 62,53,819)	−18.02% (−18,05%,−17,92%)
2022.11–2023.10	74,44,882 (74,50,230, 74,39,534)	−13.53% (−13,65%, −13,53%)	69,27,452 (69,22,293, 69,32,610)	−9.12% (−9,15%, −9,01%)

Influenza viruses—primarily influenza A subtypes H1N1 and H3N2, as well as influenza B viruses—circulate annually, though the intensity of outbreaks varies considerably from year to year. Surveillance data indicate that in certain years, the number of influenza cases rises significantly above the baseline, resulting in what is commonly referred to as a “high-flu year.” However, this phenomenon does not follow a fixed periodic pattern. While large-scale influenza outbreaks tend to occur approximately every 3–5 years, seasonal outbreaks of varying magnitude are observed each year (35, 36). In 2019, the number of influenza cases and related deaths in China reached the highest level recorded between 2012 and 2021 (35), significantly exceeding the average figures of previous years. Similar patterns were also observed in other regions, including France, New York City in the United States, and England and Wales (36–38). Therefore, we argue that the anomalous mortality data from 2019 render that year unsuitable—at least in the short term—as a baseline for adjusting or estimating age-specific excess mortality during the COVID-19 pandemic. However, we have chosen not to exclude the 2019 data from the construction of the standard age- and sex-specific baseline derived from the 2015–2019 period. This decision is based on the understanding that, over a longer time horizon, high-influenza years are not infrequent occurrences and thus should be retained to reflect realistic baseline variability.

While acknowledging the limitations of such data in providing a comprehensive comparison of excess mortality from 2020–2023, it is evident that the standard life table based on five-year data from 2015–2019 provides a more robust quantitative measure than the single-year data from 2019. This is particularly significant for capturing the contributions of medical advancements and efficacy of preventative measures to reduce excess mortality in China.

Following a thorough comparison of the available data, it was decided that the most appropriate course of action would be to analyze excess mortality data using standardized age- and sex-specific data from 2015–2019. This decision was made based on the relatively high population-based excess mortality rates observed in 2019. Due to limitations in the sample size, the excess mortality data for certain age groups exhibited relatively large fluctuations. Consequently, the decision was made to calculate the excess mortality data by age group. The infant group was separated, and the remaining ages were categorized into decade-long groups, given the inherently high mortality rate observed among 0-year-old infants and the substantial decline in infant mortality seen in the four years following the emergence of the virus. Furthermore, all age groups above the 80-year group, which represent a smaller population, were grouped together to calculate excess mortality.

Table 2 presents the excess mortality figures for the years 2020–2023, showing that the majority of age groups exhibited negative excess mortality. Notably, excess mortality figures significantly decreased for age groups above the 40-year group. However, in 2020 and 2023, excess mortality among the elderly was relatively high, indicating that despite medical advancements and the widespread use of masks, the implementation of prevention measures and vaccination rates had a considerable impact on excess mortality data.

**Table 2:** Sex- and age-standardized excess mortality of 2020–2023.

Index	Excess mortality				Excess mortality rate			
Agegroup	2020	2021	2022	2023	2020	2021	2022	2023
0	−31,133 (−31,353, −30,912)	−31,750 (−31,966, −31,535)	−32,932 (−33,136, −32,727)	−31,787 (−32,028, −31,598)	−71.03% (−71,53%, −70,52%)	−72.44% (−72,93%, −71,94%)	−75.13% (−75,60%, −74,66%)	−72.58% (−73,07%, −72,09%)
1–10	−14,062 (−14,402, −13,722)	−20,134 (−20,437, −19,830)	−12,263 (−12,613, −11,913)	−22,196 (−22,292, −21,709)	−31.87% (−32,64%, −31,10%)	−45.63% (−46,32%, −44,94%)	−27.79% (−28,59%, −27,00%)	−49.86% (−50,52%, −49,20%)
11–20	−2,654 (−3,037, −2,271)	−10,398 (−10,740, −10,057)	−7,879 (−8,235, −7,524)	−10,550 (−10,895, −10,213)	−6.51% (−7,45%, −5,57%)	−25.51% (−26,35%, −24,67%)	−19.33% (−20,20%, −18,46%)	−25.89% (−26,73%, −25,06%)
21–30	−10,273 (−10,831, −9,716)	−30,642 (−31,125, −30,160)	−39,358 (−39,805, −38,911)	−24,004 (−24,513, −23,496)	−11.25% (−11,86%, −10,64%)	−33.57% (−34,09%, −33,04%)	−43.11% (−43,60%, −42,62%)	−26.29% (−26,85%, −25,74%)
31–40	−18,724 (−19,463, −17,985)	−55,017 (−55,655, −54,379)	−63,883 (−64,493, −63,272)	−19,993 (−20,729, −19,257)	−11.64% (−12,10%, −11,18%)	−34.19% (−34,59%, −33,80%)	−39.70% (−40,08%, −39,32%)	−12.43% (−12,88%, −11,97%)
41–50	−28,993 (−30,262, −27,723)	−1,16,843 (−1,17,972, −1,15,714)	−1,05,238 (−1,06,386, −1,04,090)	−64,565 (−65,780, −63,351)	−6.46% (−6,75%, −6,18%)	−26.05% (−26,30%, −25,80%)	−23.46% (−23,72%, −23,21%)	−14.40% (−14,67%, −14,12%)
51–60	−62,320 (−64,037, −60,604)	−2,18,269 (−2,19,801, −2,16,738)	−1,31,243 (−1,32,880, −1,29,606)	−92,338 (−94,020, −90,656)	−7.52% (−7,72%, −7,31%)	−26.33% (−26,51%, −26,15%)	−15.83% (−16,03%, −15,63%)	−11.14% (−11,34%, −10,94%)
61–70	−1,27,137 (−1,29,463, −1,24,810)	−4,13,538 (−4,15,614, −4,11,462)	−1,68,247 (−1,70,539, −1,65,955)	−1,88,885 (−1,91,160, −1,86,611)	−8.28% (−8,43%, −8,13%)	−26.93% (−27,06%, −26,79%)	−10.96% (−11,10%, −10,81%)	−12.30% (−12,45%, −12,15%)
71–80	−1,28,016 (−1,30,727, −1,25,305)	−6,13,669 (−6,16,011, −6,11,327)	−3,73,334 (−3,75,865, −3,70,803)	−1,77,937 (−1,80,613, −1,75,262)	−6.27% (−6,40%, −6,14%)	−30.06% (−30,18%, −29,95%)	−18.29% (−18,41%, −18,16%)	−8.72% (−8,85%, −8,59%)
81+	−36,731 (−39,736, −33,726)	−8,02,643 (−8,05,110, −8,00,176)	−4,39,652 (−4,42,388, −4,36,917)	−63,450 (−67,631, −59,719)	−1.54% (−1,66%, −1,41%)	−33.62% (−33,72%, −33,52%)	−18.42% (−18,53%, −18,30%)	−2.66% (−2,78%, −2,53%)

To facilitate a clearer comparison of the excess mortality data, excess mortality rates across different age groups were statistically analyzed using the aforementioned categorization method. The results about excess mortality rate presented in Table 2 reveal that the most significant decline in excess mortality rates over the four-year period of 2020–2023 was observed among younger individuals. This finding suggests that, while advancements in public health and medical technology have contributed more prominently to reducing deaths among the elderly, the proportional improvement in health status has been more pronounced among younger people. Notably, the 0-year-old age group demonstrated a >70% decline in mortality between 2020 and 2023. A similar trend was observed in the 1–10 age group, which demonstrated a decrease of >25% in mortality across the four-year period. However, the reduction in mortality was comparatively lower in 2020 and 2022 than in 2021 and 2023. For the 11–20 and 21–30 age groups, the proportion of mortality reduction in 2020 was significantly lower than that in the other three years, indicating that the pandemic and associated lockdown measures had a greater impact on these two age groups in 2020. Among the seven age groups above the 20-year group, with the exception of the 61–70 age group, the majority experienced a higher reduction in mortality figures in 2022 than in 2023, suggesting that the beneficiaries of the lockdown measures instigated in response to the pandemic were primarily adults, whereas lifting these measures had a relatively small impact on minors. Moreover, while the year 2021 signified the most substantial enhancement in health status across the majority of age groups, it was observed that in the 0-year-old, 1–10-year-old, and 11–20-year-old age groups, the decline in mortality figures was more pronounced in 2023 than in 2021. This finding suggests that the lifting of lockdowns imposed owing to the pandemic had a comparatively larger impact on the health of younger age groups.

Overall, excess mortality in 2020–2023 was lower among the elderly than among the young, and among men than among women. The following discussion will focus on two points: first, a comparison between the 2022 and 2023 data to determine the current situation, and second, the differences in excess mortality rates between men and women across various age groups during the COVID-19 pandemic.

### 4.1 Comparison between excess mortality of 2022 and 2021

The excess mortality data for the years 2020–2023, estimated based on a standard life table constructed using age- and sex-specific structure dataset from the five-year period 2015–2019, reflects the substantial role played by medical improvements in reducing mortality rates. The four-year data consistently demonstrated improvements compared with the mortality data from 2015–2019, evidencing the significant health dividends brought about by advancements in medical technology and public health. Nevertheless, it should be noted that such data comparisons are unable to capture fluctuations in excess mortality resulting from changes in prevention and control policies for the virus. For instance, in 2022, China experienced several severe local outbreaks of the COVID-19 driven by variants such as Omicron BA.1, BA.2, and BA.5, resulting in prolonged lockdowns in certain regions. After the gradual relaxation of restrictions in November and December 2022, large-scale local outbreaks occurred in China. Given that these two factors are partially mitigated by medical progress, the method described in the previous section was employed to explore the impact of prevention and control policies on excess mortality. Specifically, the age and sex structure dataset from 2021 was utilized as a baseline, and the mortality data for 2022 and 2023 were adjusted accordingly. This approach enabled the generation of age- and sex-specific excess mortality data for these two years.

Based on adjusted calculations using the 2021 data as a baseline, the number of excess deaths in 2022 and 2023 were estimated to be 1,154,105 and 1,977,432, respectively, with a substantial increase of 71% from 2022–2023. Age- and sex-specific data from 2021 provide a basis for understanding the varying scales of excess deaths associated with different COVID-19 prevention and control strategies, given the latest medical advancements. Outbreaks of locally transmitted COVID-19 cases and stringent epidemic prevention policies in China by 2022 have contributed to the occurrence of excess deaths. Furthermore, the relaxation of COVID-19 restrictions has led to an even more significant surge in excess deaths.

The calculation shows that both the number of excess deaths and the excess mortality rate were significantly higher in 2022 than in 2021 and further increased significantly in 2023 compared to 2022. Table 3 shows that the excess mortality rates in 2022 are higher than those in 2021 for most age groups, especially for those aged 61 years and older. For individuals aged 0, 21–30, and 31–40 years, there was a slight decrease in mortality rates. However, there was a slight increase in mortality among children aged 1–10 years. Owing to the already high baseline mortality, the excess mortality rate for persons aged 60 years and older was not particularly high in 2022. However, compared with 2021, the majority of excess deaths in 2022 occurred in the 60 + age group. The 2023 data demonstrate the typical feature already mentioned: excess deaths in the seven age groups above the 20-year group compared with the 2021 data, and with the exception of the 61–70 age group, the excess mortality rates were higher than those in 2022.

**Table 3:** Sex- and age-standardized (by 2,021 data) excess mortality of 2022 and 2023.

Agegroup	2022	2023
0	−10.64% (−12,14% −9,06%)	0.39% (−1,25%, 2,02%)
1–10	26.93% (25,53%, 28,33%)	−13.41% (−14,38%, −12,07%)
11–20	3.88% (2,85%, 5,08%)	−2.29% (−3,37%, −1,21%)
21–30	−12.72% (−13,64%, −11,94%)	14.52% (13,54%, 15,49%)
31–40	−8.18% (−9,14%, −8,02%)	33.21% (32,38%, 33,74%)
41–50	4.37% (3,91%, 4,65%)	18.80% (18,39%, 19,18%)
51–60	13.27% (13,02%, 13,52%)	20.12% (19,86%, 20,38%)
61–70	22.76% (22,53%, 22,92%)	21.33% (21,13%, 21,52%)
71–80	17.08% (16,89%, 17,21%)	30.30% (30,13%, 30,47%)
81+	24.00% (23,85%, 24,16%)	47.34% (47,18%, 47,51%)

The excess mortality observed in the 1–10 year age group in 2022 may be related to delays in seeking healthcare. Parents avoided hospitals because of the pandemic and COVID-19 lockdown measures, which led to the inadequate management of childhood respiratory infections and chronic conditions. Conversely, the excess mortality in the 61–70 age group could be related to proactive triage by the healthcare system, where many younger or healthier elderly people are scheduled for deferred medical treatment during periods of healthcare resource shortage, contributing to a relatively higher excess mortality in this age group. Excess mortality in 2023 is mainly due to the relaxation of COVID-19 prevention and control measures in November 2022 and December 2022. The increase in COVID-19 infections over a short period of time resulted in COVID-19-related deaths and a shortage of healthcare resources, leading to excess mortality during the period from November 2022 to October 2023.

### 4.2 Sex-based differences in excess mortality rate

For further investigation, we used standardized sex- and age-specific data from 2015–2019 as the baseline to conduct a comparative analysis of the adjusted excess mortality from 2020–2023 for men and women in different age groups. Given the significant differences in mortality rates between sexes and age groups, with men and older adults typically experiencing higher mortality rates, quantifying differences in excess mortality based solely on numerical values is challenging. Therefore, we used excess mortality as a comparative measure. This metric better captured the nuances and underlying causes of the significant differences observed over the four years. We continued with the previous method of categorizing age groups, within which we calculated and compared the excess mortality rates for both men and women. This approach provides a granular understanding of how sex and age intersect with excess mortality during the pandemic, providing insights into potential disparities and their underlying causes.

Table 4 shows that in most age groups and in most years, excess mortality rates of women are comparable to those of men, with women potentially having lower excess mortality in many of the years and age groups, particularly among the elderly. In 2020, excess mortality of women was higher than that of men in the age groups 31–40 and 71–80; in particular, in the age groups 11–20, 21–30, and >81, excess mortality of women was not only higher than that of men, but also showed positive values, indicating significant excess mortality among women in these age groups in 2020. In 2021, male excess mortality continued to be higher than female excess mortality in most age groups, with a particularly large difference observed for those under 30. In 2022, excess mortality of men exceeded that of women in all age groups except in the 21–30 and 51–60 groups. Similarly, in 2023, male excess mortality was higher in all age groups except the 31–40 age group, where female excess mortality also reached positive values, alongside the positive excess mortality rates observed for men aged 81 and older. These observations suggest that in 2020, men may have benefited more from China's effective COVID-19 prevention measures, possibly because men reduced social activities during the pandemic restrictions reduced external causes of death such as traffic accidents and violent incidents. Conversely, women may have benefited more in 2021, 2022 and 2023. Given that 2021 and 2022 were the peak years of China's vaccination campaigns, it is likely that women benefited more from the vaccination programs and were less affected by the pandemic and associated containment measures.

**Table 4:** Comparison between genders on the excess mortality rate by age groups.

Agegroup	2020		2021		2022		2023	
	Male	Female	Male	Female	Male	Female	Male	Female
0	−70.88% (−71,54%, −70,21%)	−71.23% (−72,00%, −70,46%)	−70.70% (−71,37%, −70,03%)	−74.77% (−75,49%, −74,05%)	−64.56% (−65,30%, −63,83%)	−89.37% (−89,83%, −88,90%)	−80.70% (−81,24%, −80,16%)	−61.63% (−62,52%, −60,75%)
1–10	−28.23% (−29,28%, −27,18%)	−36.66% (−37,79%, −35,53%)	−24.64% (−25,72%, −23,57%)	−73.25% (−73,98%, −72,51%)	−7.45% (−8,64%, −6,26%)	−54.56% (−55,52%, −53,60%)	−41.14% (−42,09%, −40,19%)	−61.33% (−62,21%, −60,45%)
11–20	−12.52% (−13,60%, −11,44%)	8.37% (6,48%, 10,25%)	−19.09% (−20,12%, −18,05%)	−41.41% (−42,80%, −40,03%)	−7.75% (−8,85%, −6,64%)	−48.01% (−49,31%, −46,70%)	−13.21% (−14,29%, −12,14%)	−57.28% (−58,46%, −56,10%)
21–30	−15.93% (−16,62%, −15,24%)	2.24% (0,94%, 3,53%)	−27.94% (−28,57%, −27,30%)	−49.81% (−50,71%, −48,90%)	−47.85% (−48,39%, −47,31%)	−29.45% (−30,53%, −28,38%)	−18.76% (−19,44%, −18,08%)	−48.03% (−48,95%, −47,11%)
31–40	−12.60% (−13,14%, −12,07%)	−9.06% (−9,96%, −8,17%)	−31.86% (−32,33%, −31,38%)	−40.42% (−41,14%, −39,69%)	−30.44% (−30,92%, −29,96%)	−64.39% (−64,94%, −63,83%)	−22.42% (−22,92%, −21,92%)	14.22% (13,22%, 15,22%)
41–50	−1.99% (−2,34%, −1,64%)	−16.07% (−16,55%, −15,60%)	−25.67% (−25,98%, −25,37%)	−26.86% (−27,31%, −26,42%)	−18.72% (−19,04%, −18,40%)	−33.66% (−34,08%, −33,23%)	−5.41% (−5,76%, −5,07%)	−33.68% (−34,10%, −33,25%)
51–60	−6.48% (−6,73%, −6,23%)	−9.86% (−10,23%, −9,49%)	−26.48% (−26,70%, −26,26%)	−25.99% (−26,32%, −25,65%)	−16.25% (−16,48%, −16,01%)	−14.90% (−15,26%, −14,54%)	−7.93% (−8,18%, −7,68%)	−18.36% (−18,71%, −18,01%)
61–70	−5.72% (−5,91%, −5,53%)	−12.94% (−13,19%, −12,69%)	−21.76% (−21,94%, −21,59%)	−36.33% (−36,55%, −36,12%)	−6.27% (−6,47%, −6,08%)	−19.48% (−19,72%, −19,24%)	−11.18% (−11,36%, −10,99%)	−14.34% (−14,59%, −14,10%)
71–80	−6.61% (−6,78%, −6,43%)	−5.78% (−5,99%, −5,57%)	−27.11% (−27,26%, −26,96%)	−34.37% (−34,55%, −34,20%)	−13.21% (−13,38%, −13,04%)	−25.70% (−25,89%, −25,52%)	−4.02% (−4,20%, −3,85%)	−15.57% (−15,77%, −15,37%)
81+	−4.24% (−4,42%, −4,06%)	0.90% (0,72%, 1,08%)	−31.63% (−31,78%, −31,48%)	−35.41% (−35,56%, −35,27%)	−17.18% (−17,35%, −17,01%)	−19.53% (−19,69%, −19,37%)	3.39% (3,21%, 3,58%)	−8.12% (−8,29%, −7,95%)

Notably, the variation in excess mortality across age groups between the different years can be partly explained by sex. As mentioned above, the higher excess mortality rates observed in 2022 were mainly concentrated in age groups 1–10 and 11–20, with significantly higher rates for both men and women compared with 2021 and 2023. Of particular note is the positive excess mortality rate in the 2–4 age group in 2022, indicating that the number of deaths in this age group exceeded the average for the same group in the period from 2015–2019. These observations raise important questions in public health and medical research that warrant further investigation. Looking at the tables stratified by sex, within age groups 1–10 and 11–20, the excess mortality rate for women was only slightly higher in 2022 than in 2023, whereas that for men was much higher and close to zero. This suggests that the health status of men aged 1–20 years in 2022 was almost the same as the average in 2015–2019. The increase in excess mortality among those aged 40 years and older in 2022 and 2023 can be attributed to the strain on the healthcare system caused by the Delta and Omicron variants. This burden led to a shortage of healthcare workers due to infections, reduced outpatient services and bed capacity due to hospital disinfection measures, and overcrowding in hospitals and intensive care units due to an increase in COVID-19 cases. Among the seven age groups above the 20-year group, in most cases, the excess mortality rate for men was higher in 2023 than that in 2021 and 2022, except for the 61–70 age group. In the two age-defined groups above the 70-year group, the excess mortality rate for women was significantly higher in 2023 than in 2021 and 2022. This pattern can be explained by the peak of infections and shortage of healthcare resources following the relaxation of COVID-19 restrictions. As for the excess mortality rate of women aged 31–40 in 2023, based on existing literature, we hypothesize that this phenomenon may be primarily associated with the following factors: an increase in perinatal complications during pregnancy and the postpartum period; (39) a rise in maternal mortality linked to disruptions in obstetric healthcare services during the COVID-19 pandemic; (40, 41) elevated pregnancy-related risks among advanced maternal age women (≥35 years) (41–43). Studies from Sweden and Thailand have also reported increases in maternal mortality and cardiovascular-related deaths among middle-aged women during the pandemic, which may offer potential explanatory parallels for the patterns observed in China (44, 45).

## 5 Discussion

This study aimed to examine the association of age and sex with excess mortality rates. To achieve this, we collected and analyzed detailed death tolls and mortality rates from the China Statistical Yearbook of Population and Employment. We constructed a standardized sex- and age-structure dataset based on data from 2015–2019, and compared the changes in excess mortality rates of 2020–2023 among different sexes and age cohorts. The study showed a decrease in deaths in all four years between 2020 and 2023, adjusted for sex and age, compared to the expected rates from the standardized sex- and age-structure dataset. The most significant decline was observed in 2021. However, there was a rebound in deaths in 2022, indicating that the impact of the pandemic is complex and uncertain. In 2023, there was a major increase in mortality, testifying to the public health disaster that occurred when China's COVID-19 restrictions were lifted by the end of 2022. Interestingly, while older age groups exhibited relatively lower excess mortality rate, younger age groups showed lower excess mortality. Factors such as health status, exposure risk, and access to healthcare resources may have contributed to this observation in the different age cohorts.

It is noteworthy that in 2021, 2022, and 2023, excess mortality rates of men were higher than those of women, which is contradictory to the pattern observed in 2020. This finding suggests that sex differences play a significant and intricate role in influencing excess mortality during a pandemic. When comparing excess mortality data across age groups, it was found that male excess mortality rate in the 1–20 age group was higher in 2022 and 2023 than in 2021 and 2020. Additionally, the excess mortality rates for both sexes in the 40 + age groups were lower in 2021 than in 2022. These findings offer valuable insights into the causes of excess mortality during pandemics and provide new avenues for further research.

The higher number of excess mortalities in 2022 and 2023 compared to that in 2021 may be due to a combination of factors. Excess mortality is the difference between the actual number of deaths and the expected or “normal” number of deaths in a given time period. This difference may reflect the impact of various factors on population mortality, including epidemics, socioeconomic conditions, natural environment, and public health policies.

Methodologically, the comparison of excess mortality data from 2020–2023 was unaffected by standardized age and sex structure database constructed using the five year data from 2015–2019, data from 2019 alone, or adjusted data using 2021 benchmarks. However, owing to the relatively unique circumstances of 2019, characterized by higher-than-average mortality, it is not suitable for adjusting and calculating excess mortality data for the period 2020–2023. Data adjusted using the standardized age and sex structure dataset derived from the five-year data from 2015–2019 can eliminate the factors contributing to the increase in total deaths and mortality rates in China due to rapid aging and declining fertility, thus reflecting the reduction in deaths associated with medical and public health advances. Given China's significant success in COVID-19 prevention under the Zero-COVID strategy in 2021, the use of sex- and age-specific excess mortality data for 2022 and 2023, compared to 2021, allows for a better estimation of the specific magnitude of excess mortality associated with different pandemic response policies under the most recent medical and public health conditions. We argue that excess mortality in China against the backdrop of the COVID-19 pandemic and the lifting of COVID-19 restrictions should be studied using a combination of these two methods.

Despite improved disease control policies and prevention measures, new mutant strains have emerged, potentially resulting in an increased number of infections and deaths. Outbreaks continue to be significant factors in the spread of COVID-19. In 2022, China, particularly Shanghai, experienced a significant outbreak, resulting in a high number of cases, hospitalizations, and deaths due to COVID-19. This surge in cases could have overwhelmed the healthcare system owing to a shortage of resources. Additionally, the socioeconomic impact of the epidemic may have indirectly contributed to increased mortality rates. Factors, such as unemployment, poverty, and psychological stress, can increase health risks. Second, socio-economic status is an important factor that influences excess mortality. The Chinese economy is likely to be affected by the 2022 pandemic, which may lead to poor socioeconomic conditions. These conditions can affect health and mortality rates. For example, poverty, malnutrition, and inadequate healthcare resources may increase the mortality risk. It is important to note that excess mortality was not caused solely by the pandemic.

By 2022, China had adopted more effective prevention and control measures, such as vaccination and widely covered PCR tests, which helped slow the spread of the outbreak and reduce mortality. In 2020, vaccine development and distribution were still in the early stages, and most of the population had not yet been vaccinated, making them more vulnerable to the virus. However, by 2022, with the widespread production and distribution of vaccines, an increasing number of people had been vaccinated, significantly increasing the population's immunity and reducing severe illnesses and fatalities. The data from November 2022 to October 2023 clearly show that the side effects of the COVID-19 prevention measures under the Zero-COVID strategy were significantly lower than those associated with widespread viral transmission and health system burden.

However, a more nuanced discussion is required. Once COVID-19 prevention measures are lifted, it is imperative to ensure the adequate availability of healthcare resources and address the challenges faced by older people in relation to COVID-19 infection and the health system burden. Meanwhile, during the containment period necessitated by the rapid spread of the Omicron variant, which challenged the Zero-COVID strategy, special attention should be paid to the physical and mental health of adolescents, particularly males, to mitigate the adverse effects of the Zero-COVID approach on the public health system.

It is important to note that these were the only possible influencing factors. The specific causes of excess mortality need to be analyzed in the context of specific data and circumstances. Additionally, the statistics and interpretation of excess mortality data may also be affected by various factors such as data quality, statistical methodology, and sociocultural background. Therefore, caution is necessary when analyzing and interpreting the data. This study did not differentiate between the effects of epidemic control and medical crowding out. It may be challenging to accurately distinguish the respective effects of epidemic control measures (such as lockdown and travel restrictions) and healthcare system overload (strained healthcare resources that cannot meet patient needs) on the mortality rate when investigating the causes of excess mortality. These two phenomena often occur simultaneously and are interdependent, which makes it difficult to assess their effects separately. Outbreak control measures can reduce the movement of people and the risk of infection; however, they can also disrupt socioeconomic activities, with a range of indirect effects. In contrast, healthcare system overload is directly linked to the ability of patients to receive timely and effective treatment and has a direct impact on mortality. When analyzing excess mortality data, it may be difficult to completely exclude the impact of advances in medical technology and improvements in public health systems on mortality. These factors typically reduce mortality rates, but during an epidemic, their impact may be obscured by epidemic-related factors. For instance, the implementation of new treatments, creation and dissemination of vaccines, and enhancements to the public health system in response to outbreaks could potentially reduce mortality rates. However, the accurate quantification and isolation of these effects in studies can be challenging. Therefore, it is necessary to collect more comprehensive and precise data and use sophisticated modeling techniques to better reflect the situation. In addition, it is necessary to be mindful of the limitations and uncertainties of these studies and avoid overinterpreting or simplifying the results.

## Data Availability

Publicly available datasets were analyzed in this study. This data can be found here: The yearbooks are published in paper format rather than electronic format.
